# Latrocimicinae completes the phylogeny of Cimicidae: meeting old morphologic data rather than modern host phylogeny

**DOI:** 10.1186/s13071-021-04932-x

**Published:** 2021-09-03

**Authors:** Sándor Hornok, Tamara Szentiványi, Nóra Takács, Áron Botond Kovács, Olivier Glaizot, Philippe Christe, Nicolas Fasel, Miklós Gyuranecz, Jenő Kontschán

**Affiliations:** 1 Department of Parasitology and Zoology, University of Veterinary Medicine, Budapest, Hungary; 2 The Pathogen and Microbiome Institute, Northern Arizona University, Flagstaff, AZ USA; 3 Institute for Veterinary Medical Research, Centre for Agricultural Research, Budapest, Hungary; 4 Museum of Zoology, Lausanne, Switzerland; 5 Department of Ecology and Evolution, Faculty of Biology and Medicine, University of Lausanne, Lausanne, Switzerland; 6 Plant Protection Institute, Centre for Agricultural Research, Budapest, Hungary

**Keywords:** *Latrocimex spectans*, Heteroptera, Bug, Ectoparasite

## Abstract

The family Cimicidae includes obligate hematophagous ectoparasites (bed bugs and their relatives) with high veterinary/medical importance. The evolutionary relationships of Cimicidae and their hosts have recently been reported in a phylogenetic context, but in the relevant study, one of the six subfamilies, the bat-specific Latrocimicinae, was not represented. In this study the only known species of Latrocimicinae, i.e., *Latrocimex spectans*, was analyzed with molecular and phylogenetic methods based on four (two nuclear and two mitochondrial) genetic markers. The completed subfamily-level phylogeny of Cimicidae showed that Latrocimicinae is most closely related to Haematosiphoninae (ectoparasites of birds and humans), with which it shares systematically important morphologic characters, but not hosts. Moreover, in the phylogenetic analyses, cimicid bugs that are known to infest phylogenetically distant bat hosts clustered together (e.g., *Leptocimex* and *Stricticimex* within Cacodminae), while cimicid subfamilies (Latrocimicinae, Primicimicinae) that are known to infest bat hosts from closely related superfamilies clustered distantly. In conclusion, adding Latrocimicinae significantly contributed to the resolution of the phylogeny of Cimicidae. The close phylogenetic relationship between Latrocimicinae and Haematosiphoninae is consistent with long-known morphologic data. At the same time, phylogenetic relationships of genera within subfamilies are inconsistent with the phylogeny of relevant hosts.

**Supplementary Information:**

The online version contains supplementary material available at 10.1186/s13071-021-04932-x.

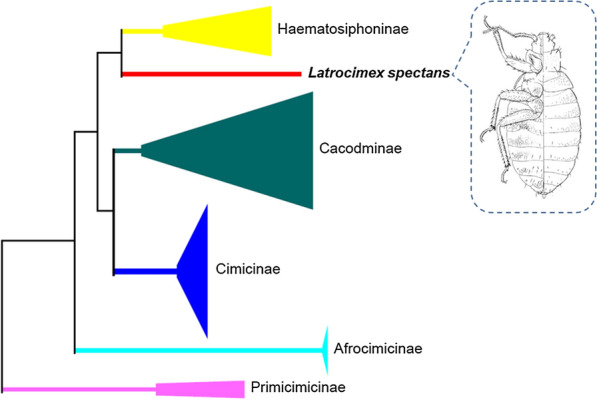

The family Cimicidae (Insecta: Hemiptera: Heteroptera) includes obligate hematophagous ectoparasites, with more than 110 described species in 24 genera and six subfamilies [1–3]. While the majority of extant cimicid species rely on bat hosts [4], several groups are adapted to other warm-blooded vertebrates, including birds and humans. In this respect, a few cimicids are generalists [3], although they have most likely evolved from host-specialist insects [5].

Even strictly host-specific permanent ectoparasites (such as blood-sucking lice) tend to show incongruent phylogenies in comparison with their hosts, probably as the result of a complex evolutionary history of host-switching events [6]. Cimicidae includes temporary ectoparasites which, in the course of their evolution, colonized bat hosts several times independently [5]. Thus, it can be expected that cimicid bugs and their bat hosts show independent phylogenetic clustering. Recently, when the host relationships of Cimicidae were analyzed in an evolutionary/phylogenetic context, one of the six subfamilies, Latrocimicinae, was not represented [5]. Here we complete the subfamily-level phylogeny of Cimicidae by adding *Latrocimex spectans* Lent, 1941, the only known species of Latrocimicinae, with *Noctilio* sp. bats as its specific hosts [1].

In this study, two male bugs were used, which were collected near the resting places of greater bulldog bats (*Noctilio leporinus*) in Belize (latitude 18.25677 and longitude −88.267594) in Central America on July 19, 2017. Both specimens were identified as *L. spectans* according to Usinger [1], and one of them was molecularly analyzed here.

The DNA was extracted from one leg of the bug with the QIAamp DNA Mini Kit (QIAGEN, Hilden, Germany) according to the manufacturer's instruction, including an overnight digestion in tissue lysis buffer and proteinase K at 56 °C.

Part of the cytochrome *c* oxidase subunit I (*cox*1) gene was amplified with a conventional PCR using the primers Lep1F (5′-ATT CAA CCA ATC ATA AAG ATA TTG G-3′), Lep1Fdeg (5′-ATT CAA CCA ATC ATA AAG ATA TNG G-3′), and Lep3R (5′-TAT ACT TCA GGG TGT CCG AAA AAT CA-3′) [7] as reported [8]. For the amplification of part of the 16S rRNA gene, the primers 16S LR-J (5′-TTA CGC TGT TAT CCC TAA-3′) and 16S LR-N (5′-CGC CTG TTT ATC AAA AAC AT-3′) were used [9, 10]. Two conventional PCRs were carried out with the primer pairs 18S-1 (5′-CTG GTT GAT CCT GCC AGT AGT-3′) and 18S-3 (5′-GGT TAG AAC TAG GGC GGT ATC T-3′), and 18S-2 (5′-AGA TAC CGC CCT AGT TCT AAC C-3′) and 18S-4 (5′-GAT CCT TCT GCA GGT TCA CC-3′) [11] to amplify approx. 1200 bp and 800 bp long fragments, respectively, of the 18S rRNA gene. In addition, the primers 1274 (5′-GAC CCG TCT TGA AAC ACG GA-3′) and 1275 (5′-TCG GAA GGA ACC AGC TAC TA-3′) [12] were used to amplify part of the 28S rRNA gene. In summary, amplified parts of the *cox*1, 16S, 18S, and 28S rRNA genes correspond to those reported in Roth et al. [5], where sequence lengths are also listed.

All above PCRs were performed in a reaction volume of 25 μl, which included 5 μl of extracted DNA and 20 μl of reaction mixture containing 1 unit HotStarTaq Plus DNA polymerase (5 U/μl) (QIAGEN, Hilden, Germany), 200 μM PCR nucleotide mix, 1 μM each primer, and 2.5 μl of 10 × Coral Load PCR buffer (15 mM MgCl_2_ included). For amplification, an initial denaturation step at 95 °C for 5 min was followed by 40 cycles of denaturation at 94 °C for 40 s, annealing at 48 °C (in case of the *cox*1 PCR at 53 °C) for 1 min, and extension at 72 °C for 1 min. Final extension was performed at 72 °C for 10 min.

Purification and sequencing of the PCR products were performed by Biomi Ltd. (Gödöllő, Hungary). Representative sequences were submitted to GenBank (*cox*1: MW269881, 16S rRNA: MW270938, 18S rRNA: MZ378786, 28S rRNA: MW270939). All those species for which sequences of all four of the above genetic markers were retrievable from GenBank according to accession numbers in Roth et al. [5] were included in the phylogenetic analyses (Additional file 1: Fig. S1). The outgroup contained all taxa from Roth et al. [5], for which all four genetic markers analyzed here were available in GenBank.

The sequences were concatenated with Geneious Prime 2019.2.3 [13] and aligned with the MAFFT algorithm [14]. A Bayesian consensus tree was created from the previously concatenated, aligned sequences using the MrBayes [15, 16] Geneious plugin, GTR (General Time Reversible) model with gamma distribution and invariant sites (GTR + G + I). The chain length was set to 5,000,000, sampling frequency to 500, and burn-in length to 100,000. The gene partitions were treated as unlinked. The random seed was set to 3967. The final phylogenetic tree was loaded into MEGAX 10.0.5 [17] for analysis. An additional phylogenetic analysis was conducted with the maximum likelihood method and Tamura–Nei model with gamma distribution and invariant sites (bootstrap: 1000) in MEGAX (10.0.5.).

The Bayesian consensus tree (Fig. 1) showed that Latrocimicinae (represented by *L. spectans*) was most closely related to Haematosiphoninae (ectoparasites of birds and humans), with strong support. These two subfamilies formed a sister group to Cacodminae and Cimicinae (Fig. 1). The topology of the maximum likelihood tree was slightly different: while the close relationship between Latrocimicinae and Haematosiphoninae was confirmed, these two belonged to a sister group of Cimicinae (Fig. 2). In comparison with previous findings [5], the above results imply that by adding Latrocimicinae, the sister group position of Cacodminae and Haematosiphoninae changed.

**Fig. 1:**
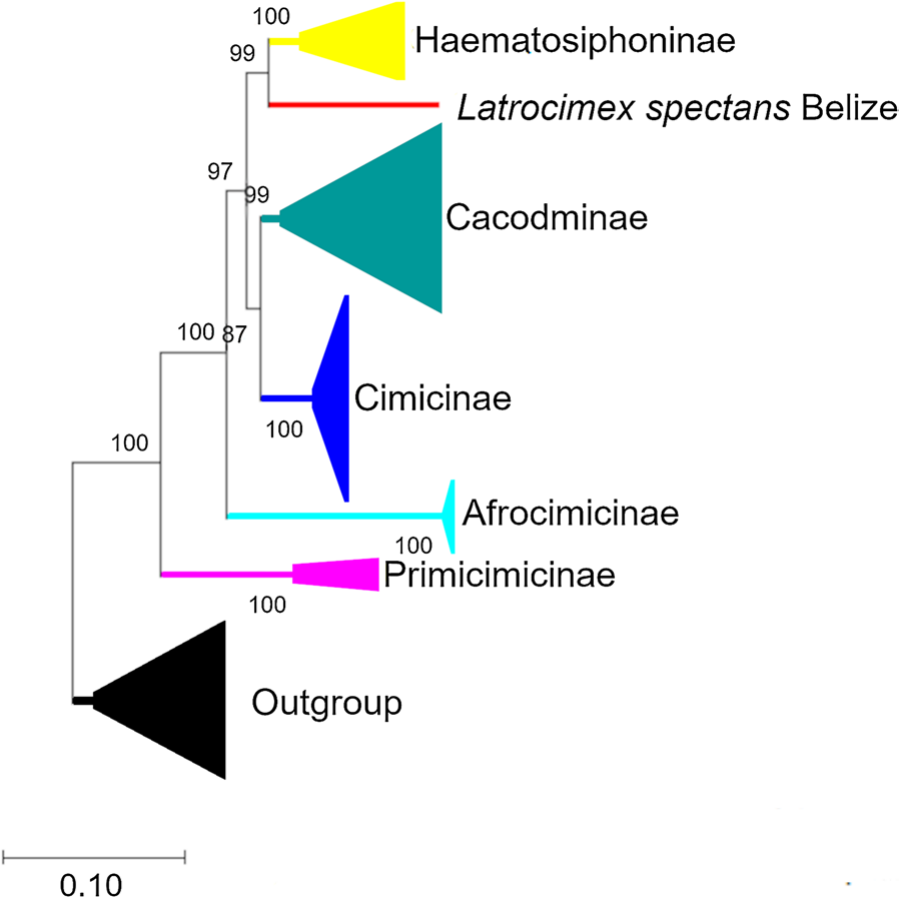
Bayesian consensus tree of Cimicidae (including all six subfamilies) based on concatenated sequences of the cytochrome *c* oxidase subunit 1 (*cox*1), 16S, 18S, and 28S rRNA genes. Cimicid subfamilies are collapsed for better overview. The outgroup contained all taxa from Roth et al. [5], for which all four genetic markers analyzed here were available in GenBank. The scale bar indicates the number of substitutions per site

**Fig. 2:**
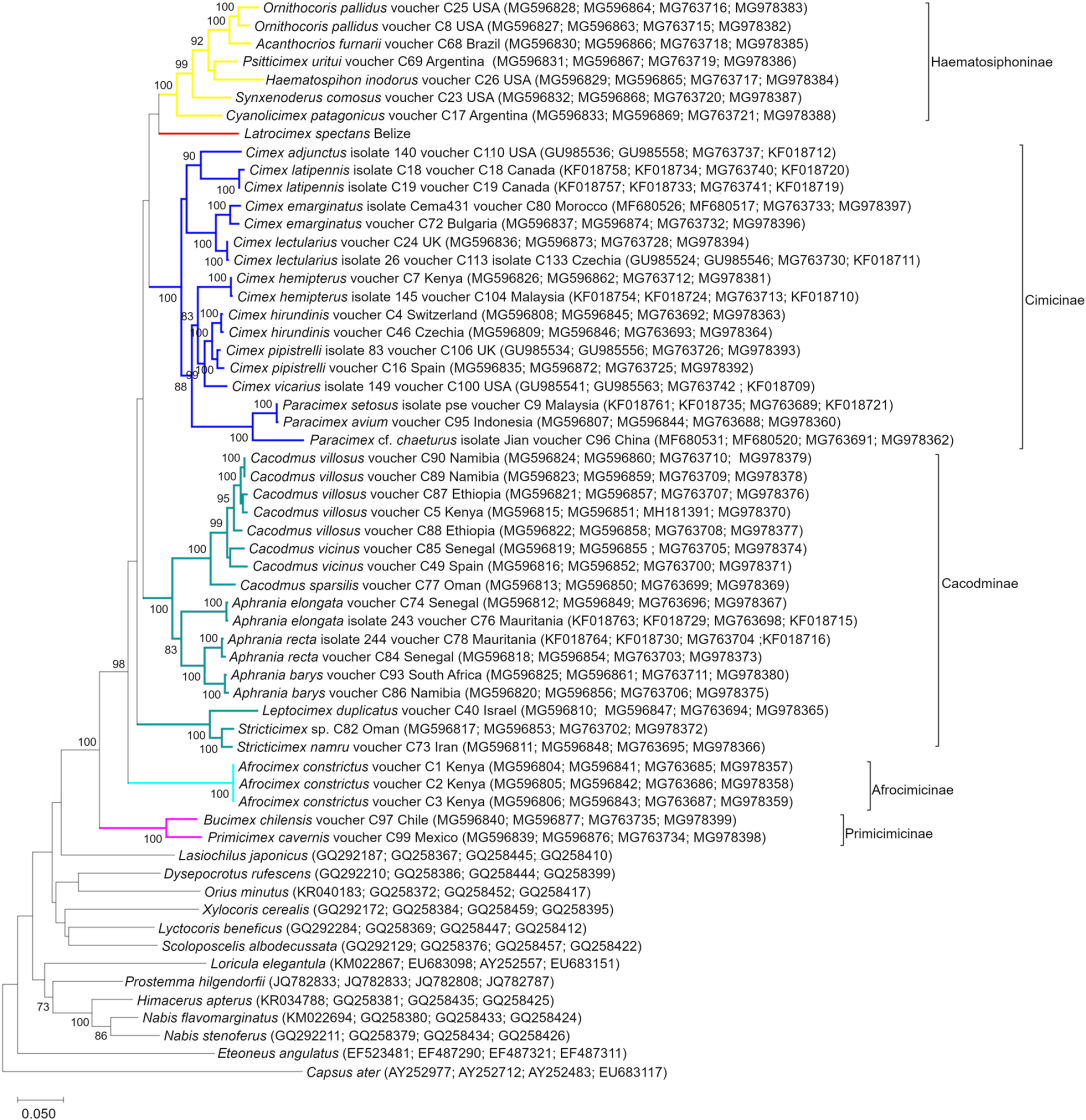
Maximum likelihood tree from concatenated sequences of the cytochrome *c* oxidase subunit 1 (*cox*1), 16S, 18S, and 28S rRNA genes. GenBank accession numbers are shown in parentheses. The phylogenetic tree was made with the Tamura–Nei model (with gamma distribution and invariant sites), based on 1000 bootstrap resamplings. The scale bar indicates the number of substitutions per site

Considering the hitherto enigmatic position of Latrocimicinae, these results confirm the morphologic tree [18] which was based on the relationships proposed by Usinger [1]. In particular, the genus *Latrocimex* belonged to the same cluster with (and next to) Haematosiphoninae, forming a sister group to Cimicinae together with Cacodminae [18]. Usinger [1] already noted that Latrocimicinae and Haematosiphoninae share systematically important morphologic characters, such as the prominent bristles on the hind angles of the pronotum, or the stout, spine-like bristles on the tibiae, as well as the right-ventral position of the paragenital sinus.

By contrast, when comparing *Latrocimex* with *Afrocimex*, it was mentioned that the number of autosomes is the same, but the two groups are completely unlike in appearance and therefore probably have no close connection [1]. This is also well reflected by the concatenated phylogenetic tree (Fig. 1), on which the two subfamilies, Afrocimicinae and Latrocimicinae, are only distantly related. Thus, the above morphologic characters are in line with the results of phylogenetic analyses performed here when including Latrocimicinae.

On the other hand, completing the phylogenetic tree with Latrocimicinae also highlights inconsistencies between host and parasite phylogenies. For instance, regarding bug genera with bats as typical hosts, the two distantly related cimicid subfamilies, Latrocimicinae and Primicimicinae, have hosts from two bat superfamilies (Noctilionoidea and Vespertilionoidea, respectively) [1] which are closely related (sister groups: [19]). At the same time, closely related cimicid genera such as *Leptocimex* and *Stricticimex* (Fig. 2) have bat hosts [1] from the most distantly related bat superfamilies Vespertilionoidea, Emballonuroidea, and Rhinolophoidea [19].

Regarding geographical ranges on the subfamily level, the ancestral Primicimicinae is Nearctic-Neotropical, whereas the core of the phylogenetic tree is occupied by Old World groups, as well as by Cimicinae showing global distribution (Fig. 1). The sister groups Latrocimicinae and Haematosiphoninae both occur in the New World. This geographically consistent pattern might reflect that the temporary parasitism by cimicid bugs prevented them from frequent natural dispersal events, especially on a transcontinental scale [3]. Accordingly, while their hosts are highly mobile, cimicid populations tend to be genetically more isolated [20] (except for the common bed bug, *C. lectularius*, and the tropical bed bug, *C. hemipterus*, which became cosmopolitan in distribution as the result of artificial dispersal events, owing to their association with humans and man-made objects).

In summary, the complete phylogeny of Cimicidae reflects morphologic relationships, rather than host associations. However, the latter might be more relevant if approached in a geographical context.

## Supplementary Information

**Additional file 1: Figure S1**. The original, detailed phylogenetic tree corresponding to Fig. 1. GenBank accession numbers are shown in parentheses.

## Data Availability

The sequences obtained and/or analyzed during the current study are deposited in GenBank (cox1: MW269881, 16S rRNA: MW270938, 18S rRNA: MZ378786, 28S rRNA: MW270939). All other relevant data are included in the manuscript and the references or are available upon request from the corresponding author.

## Abbreviation

*cox*1: Cytochrome *c* oxidase subunit 1
