# Supplementary figures and images for: Latrocimicinae completes the phylogeny of Cimicidae: meeting old morphologic data rather than modern host phylogeny
Source: Parasit Vectors. 2021 Sep 3;14:441. doi: 10.1186/s13071-021-04932-x (PMC8414776; doi:10.1186/s13071-021-04932-x)

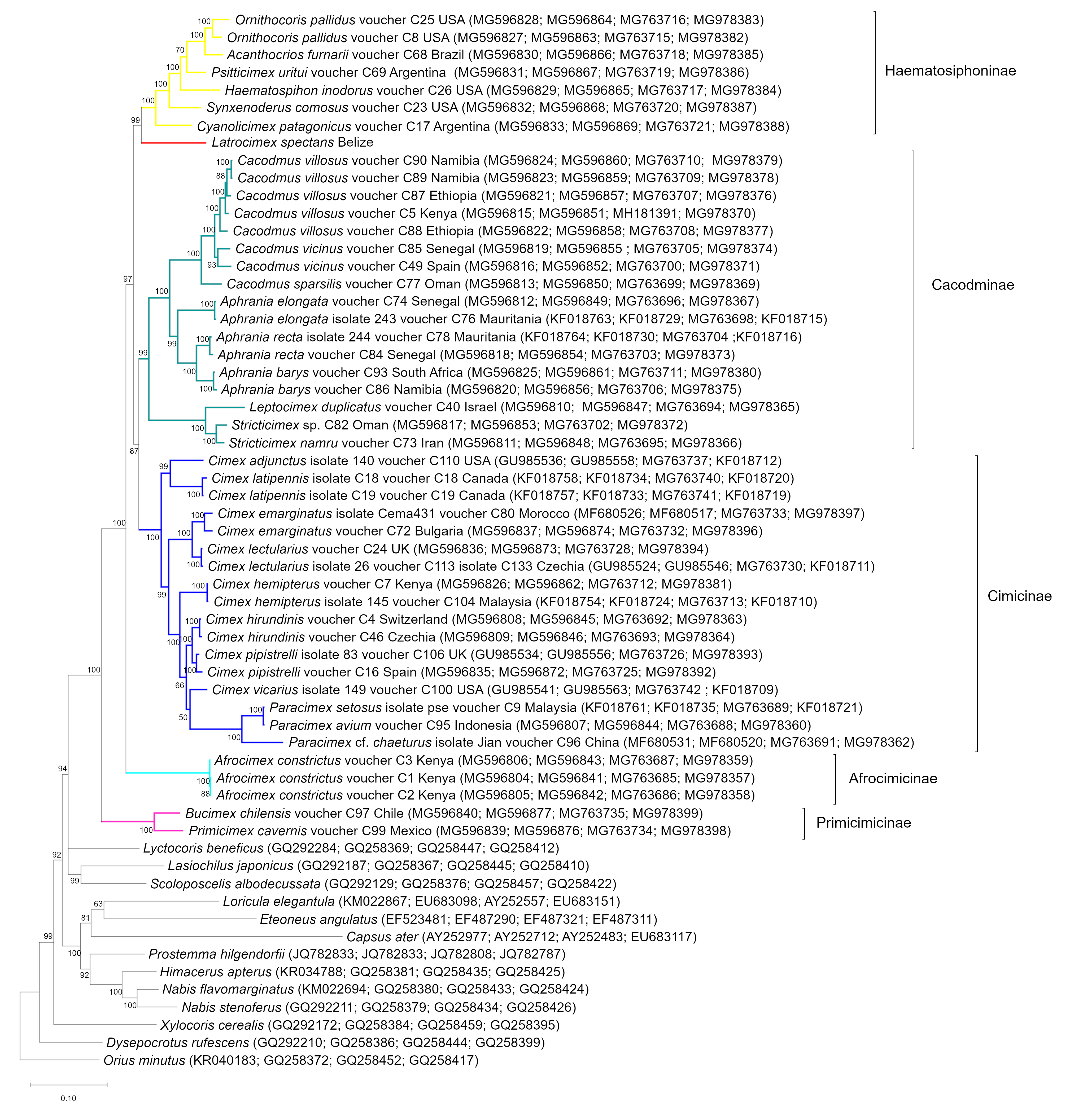

Supplement: Supplementary file 1 — Additional file 1: Figure S1. The original, detailed phylogenetic tree corresponding to Fig. 1. GenBank accession numbers are shown in parentheses. [file 13071_2021_4932_MOESM1_ESM.png]
